# Semi-Blind Signal Extraction for Communication Signals by Combining Independent Component Analysis and Spatial Constraints

**DOI:** 10.3390/s120709024

**Published:** 2012-07-02

**Authors:** Xiang Wang, Zhitao Huang, Yiyu Zhou

**Affiliations:** College of Electronic Science and Engineering, National University of Defense Technology (NUDT), Changsha 410073, China; E-Mails: taldcn@sohu.com (Z.H.); zhouyiyu@sohu.com (Y.Z.)

**Keywords:** independent component analysis (ICA), constrained ICA, signal extraction, spatial constraint, initialization

## Abstract

Signal of interest (SOI) extraction is a vital issue in communication signal processing. In this paper, we propose two novel iterative algorithms for extracting SOIs from instantaneous mixtures, which explores the spatial constraint corresponding to the Directions of Arrival (DOAs) of the SOIs as a priori information into the constrained Independent Component Analysis (cICA) framework. The first algorithm utilizes the spatial constraint to form a new constrained optimization problem under the previous cICA framework which requires various user parameters, *i.e.*, Lagrange parameter and threshold measuring the accuracy degree of the spatial constraint, while the second algorithm incorporates the spatial constraints to select specific initialization of extracting vectors. The major difference between the two novel algorithms is that the former incorporates the prior information into the learning process of the iterative algorithm and the latter utilizes the prior information to select the specific initialization vector. Therefore, no extra parameters are necessary in the learning process, which makes the algorithm simpler and more reliable and helps to improve the speed of extraction. Meanwhile, the convergence condition for the spatial constraints is analyzed. Compared with the conventional techniques, *i.e.*, MVDR, numerical simulation results demonstrate the effectiveness, robustness and higher performance of the proposed algorithms.

## 1. Introduction

The problem of blind source separation arises in a wide range of application fields, such as speech processing [1], image analysis [2], medical diagnosis [3] and wireless communication [4], *etc.* Suppose that there exist *M* independent source signals **s**(*n*) = [*s*_1_(*n*), *s*_2_(*n*), …, *s_M_*(*n*)]*^T^* and *N* observed mixtures of the source signals **x**(*n*) = [*x*_1_(*n*), *x*_2_(*n*), …, *x_N_*(*n*)]*^T^*. The linear instantaneous model of Blind Source Separation (BSS) is as follows:

(1) x(n)=As(n)+v(n)

where **A** is an *N* × *M* mixing matrix and **v**(*n*) = [*v*_1_(*n*), *v*_2_(*n*), …, *v_N_*(*n*)]*^T^* denotes the noise vector. The goal of BSS is to determine the original sources **s**(*n*) from their mixtures **x**(*n*) without any other a priori knowledge being necessary.

However, some applications of BSS often wish to extract only one signal of interest (SOI) or a desired subset of sources and automatically discard uninteresting sources: for example, the extraction of “interesting” signal from the interference signals in communication application. In such cases, the BSS problem reduces to a blind signal extraction (BSE) problem.

Generally speaking, two kinds of promising techniques have been proposed to address the BSE problem. One is beamforming, which attempts to cover specific cell sectors so that the signal of interest (SOI) can be extracted while suppressing other signals. Traditional beamforming techniques such as MVDR, LCMV [5] are based on the accurate knowledge of the direction vector associated to the SOI and the perfect array calibration, both of which are not often available in practice. Another technique is independent component analysis (ICA), which is perhaps most widely used for performing BSS. ICA attempts to exploit the assumed mutual statistical independence of the source components to estimate the mixing matrix and/or the source signals which can have an arbitrary permutation of the original sources [6]. Many existing ICA algorithms recover the source signals simultaneously whose number is same as that of the observed mixtures, while in many practical applications, it is not necessary for us to extract all the source signals since the number of desired signals needed to be recovered is less than that of the mixtures. Therefore, classical ICA algorithms involve redundant computation, require large memory for estimating uninteresting signals, degrade the quality of the signals recovered and need complex post-processing to detect and identify sources of interest. Furthermore, Hiroshi *et al.* proposed a new algorithm [7,8] combing subband ICA and beamforming to solve convolutive blind source separation problem in frequency domain, which not only helps to solve arbitrariness of permutation and gain problem at each frequency bin, but also improve the separation performance. In [9], the geometric source separation (GSS) algorithm, which combines the optimization criteria of source separation, while constraining the responses of multiple beams based on readily available geometric information, can be used to extract the SOIs while reducing undesired interferences. However, it is a symmetric algorithm which recovers the source signals simultaneously whose number is same as that of the observed mixtures, though often in the BSE problem, especially when the number of components needed to be recovered is much less, the one-unit (deflation) scheme is recommended. Although some one-unit algorithms were proposed to extract all source signals one by one with a deflation process, the inefficiency of such techniques and the arbitrary order of extraction remain as major drawbacks. Elimination of indeterminacy in the ICA not only resolves the aforementioned problems but facilitates more applications by providing stable and unique solutions.

Recently, Lu and Rajapakse proposed a new technique of constrained independent component analysis (cICA) [10], which incorporates the *a priori* information as additional constraints into the conventional ICA learning process and means that only a single statistically independent component will be extracted for each given constraint, which can help to reduce the dimensionality of the output of the ICA method. The *a priori* information in [11,12] are rough templates or reference signals of the desired signals, and the constraints are denoted by the correlation measure between the recovered signals and their corresponding reference signals. James *et al.* have applied with great success the cICA method to artifact rejection in EEG/MEG signal analysis [13]. Lee *et al.* used the cICA method for the extraction of fetal abdominal ECGs [3]. In practice, it is difficult for us to accurately compensate the time delay between the recovered signal and the reference signal in order to make the phase between them be closely matched, let alone that the reference signals are not available in communication applications Mitianoudis *et al.* [14] proposed a new cICA method exploiting the smoothness constraint to extract the smooth source signals with slowly varying temporal structures. Furthermore, the previous cICA methods generally view the *a priori* information as inequality constraints and transform the BSE problem into a constrained optimization problem. It means that the *a priori* information has been incorporated into the learning process to guarantee the algorithms converge to the desired solution. Yet, all of these algorithms need a very important parameter, e.g., closeness tolerance in [11–13] and smoothness degree in [14], to measure the corresponding constraint. Unfortunately, it is not easy for us to obtain a suitable candidate, which needs a great deal of trial.

We notice that, in communication applications with antenna arrays, the mixing matrix is closely related to the Direction of Arrivals (DOAs) of the narrowband source signals and the advantage of the beamforming technique over the source separation method lies in its use of geometric information. The a priori information about the structure of selected source sensor projections is often readily available and can similarly be used as a reference or spatial constraint, which is associated with the DOA of the SOI as well as in the beamforming theory. This paper is concerned with the use of spatial constraints in the cICA method and aims to introduce a novel method by incorporating the rough spatial knowledge into the initialization of the ICA method, instead of its learning process. Our purpose is to describe how the computationally fast and efficient, the ICA algorithm can be adapted to accommodate spatial constraints and thus provide an improved algorithm. This manuscript is organized as follows: in Section 2, the attenuation delay mixing model is given, along with the assumptions and the notations. Section 3 briefly reviews the ICA concept and the cICA framework. Section 4 introduces how the spatial constraint can be incorporated into the ICA process and demonstrates its efficacy in extracting the SOI. Section 5 presents the results of a computer simulation compared with the beamforming method and finally Section 6 provides the conclusions and discussion.

## 2. Problem Formulation, Definition, Assumption and Notation

### 2.1. Notation

Conventional notation is used in this paper. Scalars, matrices and vectors are represented by lower case, upper case and boldface lower case letters, respectively. The i^th^ component of vector **x** is denoted by *x_i_*. The expectation operator is E{·}, **A**^T^, A* and **A**^H^ denote transpose, complex conjugate, and Hermitian transpose of the matrix **A**, respectively. The identify matrix is denoted by **I**. Furthermore, *kurt*(·) denotes the kurtosis operator and ‖·‖ represents the L2 norm of a vector.

### 2.2. Problem Formulation

In narrowband (NB) array signal processing, the attenuation delay mixing model is more suitable than the instantaneous mixing model. Suppose *N* narrowband source signals impinge on a linear array of *M* sensors, the i^th^ mixture *x_i_*(*n*) can be formulated as:

(2) $$x_{i}(n)=\sum_{k=1}^{N}b_{ik}s_{k}(n-\tau_{ik})+v_{i}(n)$$

where *b_ik_* is the attenuation coefficients, and *τ_ik_* denotes the propagation time delays associated with the path from the k^th^ source signal to the i^th^ sensor which can be represented by *c*^−1^*d_i_*sin*θ_k_* where *d_i_*, *c* and *θ_k_* denote the position of the i^th^ sensor, the propagation velocity and the DOA of the k^th^ source signal, respectively. According to the NB assumption, Equation (2) can be formulated as complex-valued form:

(3) $$x_{i}(n)=\sum_{k=1}^{N}b_{ik}s_{k}(n)e^{-j\pi f_{k}c^{-1}d_{i}\sin\theta_{k}}+v_{i}(n)$$

Equation (3) can be rewritten as the matrix form (1), where the mixing matrix can be represented by the DOAs of the source signals **A** = [**a**_1_(*θ*_1_), …, **a***_N_*(*θ_N_*)]*^T^* ∈ ∁^*M* × *N*^. For instance, in a uniform linear array (ULA) system where the inter-element equals to half of the wavelength of the source signal, the steering vectors in the mixing matrix can be denoted by **a***_k_*(*θ_k_*) = [1, ***e***^‐^*^jπsinθk^*, …, ***e***^‐^*^j^*^(^*^M^*^‐1)^*^πsinθk^*]*^T^* ∈ ∁^*M* × 1^. The model throughout this paper is a uniform linear array.

### 2.3. Spatial Constraints on the Mixing Matrix

Given the *a priori* knowledge on the rough estimate of DOAs **θ̂** = [*θ*_1_, *θ*_2_, …, *θ_l_*]*^T^* of the SOIs, we can define a spatial constraint on the mixing matrix, *i.e.*, the spatial constraint regarding the sensor projections of the SOIs operates on selected columns of **A** and are enforced with reference to a set of predetermined constraint sensor projections, denoted by **A***_c_*. Thus, the spatially constrained mixing matrix comprises two types of columns [15]:

(4) $$A=\left[{\hat{A}}_{c}A_{u}\right]$$

where **Â***_c_* ≈ **A***_c_* are columns subject to the constrained and can be written as follows:

(5) $$\begin{array}{cc} {\hat{a}}_{i}=\left[\begin{array}{cccc} 1 & e^{-j\pi {\hat{f}}_{i}c^{-1}d_{2}\sin{\hat{\theta }}_{i}} & \ldots & e^{-j\pi {\hat{f}}_{i}c^{-1}d_{N}\sin{\hat{\theta }}_{i}} \end{array}\right] & i=1,2,\cdots ,l \end{array}$$

and **A***_u_* are otherwise unconstrained columns.

To reflect the degree of certainty about the accuracy of the constrained topographies **A***_c_* and the extent to which **Â***_c_* may diverge from **A***_c_*, we make the definition of the inverse of the mixing matrix as follows:

(6) $$A^{-1}=\left[{\tilde{a}}_{1}^{H}{\tilde{a}}_{2}^{H}\cdots {\tilde{a}}_{N}^{H}\right]$$

In this manuscript, the accurate degree of the constrained column **â***_i_* for the desired signal *s_i_*(*n*) contrast with sensor projections for other signal *s_j_*(*n*) is defined as follows:

(7) $$\begin{array}{cc} AD\left({\hat{a}}_{i}|{\tilde{a}}_{j}\right)=\left|\frac{{\tilde{a}}_{i}^{H}{\hat{a}}_{i}}{{\tilde{a}}_{j}^{H}{\hat{a}}_{i}}\right| & j=1,2,\cdots ,Nj \end{array}\neq i$$

### 2.4. Assumption

In our implementation of cICA with the spatial knowledge, we make assumptions that are in keeping with the general assumptions governing the application of ICA. In particular, we assume the following:

**AS1:** All the source signals are independent from each other.
  In practice, this assumption is not strict and easy to satisfy.
**AS2:** The SOI is not Gaussian.
  Most digital communication signals can be considered as sub-Gaussian and therefore this assumption is also within reason.
**AS3:** The number of sensors is assumed to be identical to that of source signals for simplicity and the mixing matrix A is of full-rank.
  This assumption is necessary for preventing the BSS problem from becoming an underdetermined case which requires other separation methods. As long as there are no two signals whose frequencies and DOAs equal to each other completely at the same time, the mixing matrix can be considered as full-rank.
**AS4:** The accuracy degree of each spatial reference or constraint satisfies the following condition:
  (8) $$\begin{array}{cc} AD\left({\hat{a}}_{i}|{\tilde{a}}_{j}\right)>\max\left(1,|{\left\|\frac{\text{kurt}(s_{j})}{\text{kurt}(s_{i})}\right\|}^{1/2}\right) & j=1,2,\cdots ,Ni\neq j \end{array}$$
  Remark: The accuracy degree required in the following analysis is related to the “non-Gaussianity” of the source signals and the mixing vectors corresponding to other “uninteresting” signals. Here, we use kurtosis as the measurement of non-Gaussianity of the source signals for simplicity.

## 3. Constrained Independent Component Analysis

### 3.1. Independent Component Analysis

As ICA is a building-block in the cICA algorithm, we start with a short description. ICA is a statistical method for transforming an observed multidimensional random vector into components that are statistically as independent from each other as possible. Thus the starting point for ICA is the very simple assumption that the components (called source signals in BSS) are statistically independent. ICA attempts to find an *N* × *M* demixing matrix **W** to recover source signals as follows:

(9) $$\hat{s}(n)=Wx(n)=\text{WAs}(n)=\text{PDs}(n)$$

where **P** ∈ ∁^*N* × *N*^ is a permutation matrix, and **D** ∈ ∁^*N* × *N*^ is a diagonal scaling matrix. Consequently, the source signals are recovered up to scaling and permutation ambiguities. In the past decade, a variety of ICA algorithms based on different methodologies and theories have been widely studied, like Bell and Sejnowski's infomax algorithm [16], Cardoso's joint approximate diagonalization of eigen matrices (JADE) [17], Hyvärinen's fixed point algorithm [18,19], *etc.* (for more recent work see [20–22]). We will restrict ourselves to the exploitation of the FastICA algorithm, mainly because of its ease of implementation and speed of separation. The FastICA algorithm is a two step procedure where the mixture data are whitened in the first step, and in the next step, unitary separate vector is updated in order to produce independent output components. Whitening means that the original observed data **x** is linearly transformed to vectors **z** = **Vx**, such that the correlation matrix of **z** equals unity: *E*{**zz***^H^*} = ***I***, which can reduce the search for an extracting vector to the group of vectors with unity norm and orthogonal to each other. This transformation can be accomplished by a principle component analysis (PCA) step. The FastICA algorithm separates the source signals based on the “non-Gaussianity” of the recovered source signals, which is measured by kurtosis or negentropy. In the FastICA algorithm for complex valued signals, the flexible and reliable approximation of negentropy was introduced in [19] as follows:

(10) $$J_{1}(w)=E\left\{G\left({\left|w^{H}z\right|}^{2}\right)\right\}$$

where *G*(·) is a non-quadratic function.

### 3.2. CICA Framework

cICA can eliminate the indeterminancy of classical ICA on permutation and consequently get the unique result by incorporating the additional requirements and available *a priori* information [10]. The general framework of cICA can be expressed as follows:

(11) $$\begin{array}{ll} \underset{w}{\max} & J_{1}(w)\\ s.t. & J_{2}(w)\leq 0\\ & J_{3}(w)=0\\ & J_{4}(w)={\left\|w\right\|}^{2}-1=0 \end{array}$$

where *J*_1_(**w**) is the contrast function which can be defined by the independence between estimated signals, while *J*_2_(**w**) = [*J*_21_(**w**), …, *J*_2_*_m_*(**w**)]*^T^* and *J*_3_(**w**) = [*J*_31_(**w**), …, *J*_3_*_n_*(**w**)]*^T^* are inequality and equality constraints denoting the available *a priori* information which of course can be combined into the contrast function or exploited in the initialization of the solution vector [23] as described in Section 4.2. Therefore, *J*_2_(**w**) and *J*_3_(**w**) are not often necessary according to different application. *J*_4_(**w**) indicates constrains the norm of extracting vector **w** to be unity due to the fact that the variance of recovered sources must be constrained to unity for whitened data.

## 4. The Proposed Algorithm with Spatial Constraint

Firstly, we derive a new cICA algorithm with the spatial constraint by using the gradient ascent method and then we will propose a novel method by incorporating the spatial knowledge into the initialization of the extracting vector instead of the learning process. Therefore, no extra parameters are involved in the algorithm, which is superior to the previous algorithms.

### 4.1. Conventional Approach with Spatial Constraint

If the minimum of half the wavelength of all source signals is longer than the sensor spacing, there is no spatial aliasing. In most such cases, the desired solution **w***_i_* forms spatial nulls in the directions of jammer signals and extracts the SOI in another direction [5]. Thus, the extraction system for the SOI *s_i_*(*n*) can be viewed as an impulse response from the mixtures to its estimate *ŝ_i_*(*n*), which can be written as follows:

(12) $$u_{i}(\theta_{i})=w_{i}^{H}h_{i}=w_{i}^{H}(Va_{i})=\sum_{j=1}^{N}w_{ij}^{*}\sum_{k=1}^{N}v_{ik}e^{-j\pi (k-1)\sin\theta_{i}}$$

In beamforming theory, the impulse response is thus called a directivity pattern, which can measure the spatial constraint. If the i^th^ row in the separating matrix produces the “interesting” source signal originating from the direction *θ_i_*, it should maximize the gain of |*u_i_*(*θ_i_*)|, that is:

(13) $$\begin{array}{cc} \left|w_{i}^{H}V{\hat{a}}_{i}\right|>\gamma >\left|w_{k}^{H}V{\hat{a}}_{i}\right| & k=1,2,\cdots ,Nk\neq i \end{array}$$

where *γ* is a threshold measuring accuracy degree of the spatial constraint. Thus, an inequality constraint can be defined for the desired output component with the directivity pattern at *θ_i_* more than or equal to a threshold *γ*, that is:

(14) $$J_{2}(w)=\gamma -\left|w^{H}V{\hat{a}}_{i}\right|\leq 0$$

Similarly, the problem of spatial constrained ICA can be modeled in the cICA framework as a constrained optimization problem:

(15) $$\begin{array}{ll} \underset{\text{w}}{\max} & J_{1}(\text{w})\\ s.t. & J_{2}(w)=\gamma -\left|w^{H}V{\hat{a}}_{i}\right|\\ & J_{4}(w)={\left\|w\right\|}^{2}-1=0 \end{array}\leq 0$$

Firstly, we replace the inequality constraint by the equality constraint max(*J*_2_(**w**), 0) = 0 for simplicity and then a neural algorithm using the augmented Lagrange multipliers method and the gradient ascent learning approach can be derived to obtain the desired optimal solution. The corresponding Lagrangian function *L*(**w**, *λ*) is given by:

(16) $$L(w,\lambda )=J_{1}(w)-\lambda \max|\left(J_{2}(w),0\right)$$

The unit-norm constraint in Equation (15) is enforced by the projection of the estimated **w** on the unit-sphere in each iteration, that is:

(17) w=w/‖w‖

Following the strategy proposed in [24], the above optimization problem is addressed using alternative optimization. That is, given the current estimate *λ*, a new estimate for **w** is searched, and then given the estimated for **w**, we update *λ*. The cICA algorithm reported here search for the optimal solution of **w** and the Lagrangian parameter *λ* by using conventional gradient descent for complex variable [24]:

(18) $$\begin{array}{l} \text{w}\leftarrow \text{w}+\eta_{1}\nabla_{\text{w}}L\\ \lambda \leftarrow \lambda -\eta_{2}\max(J_{2}(w),0) \end{array}$$

where *η*_1_, *η*_2_ are the corresponding learning rate and ∇**_w_***L* denotes the gradient vector of *L*(**w**, *λ*) (See the Appendix I):

(19) $$\nabla_{w}L=\text{E}\left\{z{\left(w^{H}z\right)}^{*}g\left({\left|w^{H}z\right|}^{2}\right)\right\}+\frac{\lambda }{2}(\text{sign}(J_{2})+1)\left[V{\hat{a}}_{i}{\left(w^{H}V{\hat{a}}_{i}\right)}^{*}\right]$$

where *g*(**·**) denotes the first-order derivative of *G*(**·**). In fact the selection of the learning rate (step size) is a crucial point and it has been a research focus for several decades where various step size selection schemes have been developed (see for example [25,26]).

If there is a subset of SOIs with the same spatial constraint, we need to run the same procedure by re-initializing the extracting vector **w***_i_* in order to identify the whole subset of SOIs. To prevent different vectors from converging to the same independent component, we must decorrelate the outputs $w_{1}^{H}z$, ${\text{w}}_{2}^{H}\text{z}$, … As introduced in [6], there are two varieties of the FastICA algorithm: the one-unit, or deflation algorithm and the symmetric algorithm. So does our algorithm for extracting a desired subset of SOIs with the same cyclic frequencies. The one-unit approach estimates the source signals successively under orthogonality condition, while the symmetric algorithm estimates all the source signals in parallel and each step is completed by a symmetric orthogonalization of the extracting matrix. In the one-unit approach, the i^th^ extracting vector **w***_i_* can be orthogonal to the space spanned by the vector **w**_1_, **w**_2_, …, **w***_i_*_-1_, by Gram-Schmidt method, that is $w_{i}=w_{i}-\overset{i-1}{\underset{j=1}{\text{∑}}}\left(w_{j}^{H}w_{i}\right)w_{j}$.

In the symmetric algorithm the symmetric orthogonalization procedure can be approximately finished by (**WW***^H^*)^−1/2^**W**. Therefore, the one-unit and symmetric version of the proposed algorithm with spatial constraint are summarized in Algorithms 1 and 2 respectively. We refer them to as Alg 1 and Alg 2 in the later analysis for simplicity.


**Algorithm 1.** The one-unit extracting algorithm with spatial constraint.
**Initialization**Whitened the observation data **x** to give **Z** = **Vx**;**for*P*** = **1, …,*l***Set *λ*^(0)^, *η*_1_, *η*_2_ and choose a random initial weight vector **w^(0)^** with unity norm**Iteration**At the i^th^ iteration for obtaining **W***_p_*,Calculate **Δ**_w_***ζ*** according to Equation (19) by utilizing **W***_p_***^(^***^i^*^−1^**^)^** respectively
$w_{p}^{\left(i\right)}\leftarrow w_{p}^{\left(i-1\right)}+\eta_{1}\nabla_{w}\zeta \left(w_{p}^{\left(i-1\right)}\right)$
$w_{p}^{\left(i\right)}\leftarrow w_{p}^{\left(i\right)}-\overset{p-1}{\underset{j=1}{\text{∑}}}w_{j}^{H}w_{p}^{\left(i\right)}w_{j}$
$w_{p}^{\left(i\right)}\leftarrow w_{p}^{\left(i\right)}/\left\|w_{p}^{\left(i\right)}\right\|$
$\lambda^{\left(i\right)}\leftarrow \lambda^{\left(i-1\right)}-\eta_{2}\max\left(J_{2}\left(w_{p}^{\left(i\right)}\right),0\right)$**Termination**The iteration is terminated when the relative change ‖**w***_p_*^(^*^i^*^)^-**w***_p_*^(^*^i^*^−^*^1^*^)^‖ is less than a specified tolerance.**end for**

**Algorithm 2.** The symmetric extracting algorithm with spatial constraint.
**Initialization**Whitened the observation data **x** to give **z** = **Vx**;Set *λ***^(0)^**, *η*_1_, *η*_2_ and choose a random initial weight matrix **W^(0)^** = |**w**_1_^(0)^, …, **w***_l_*^(0)^|with **w***_l_*^(0)^ having unity norm**Iteration**At the i^th^ iteration for obtaining **W**,**for**
*P* = 1, …, *l*Calculate ∇**_w_**ζ according to Equation (19) by utilizing 
$w_{p}^{\left(i-1\right)}$ respectively
$w_{p}^{\left(i\right)}\leftarrow w_{p}^{\left(i-1\right)}+\eta_{1}\nabla_{w}\zeta \left(w_{p}^{\left(i-1\right)}\right)$**end for**
$w^{\left(i\right)}\leftarrow {\left(w^{\left(i\right)}{\left(w^{\left(i\right)}\right)}^{H}\right)}^{-1/2}w^{\left(i\right)}$
$\begin{array}{cc} \lambda^{\left(i\right)}\leftarrow \lambda^{\left(i-1\right)}-\eta_{2}\max\left(\min\left(J_{2}\left(w_{p}^{\left(i\right)}\right)\right),0\right) & p=1,\cdots ,l \end{array}$**Termination**The iteration is terminated when the relative change ‖**W**^(^*^i^*^)^ − **W**^(^*^i^*^−1)^‖ less than a specified tolerance.


Yet Alg1 and Alg2 require a user parameter which may affect the final results significantly. The selection of the threshold in the algorithm is of vital importance for extracting the desired signal successfully, which can be found in Section 5 by simulation. Furthermore, the update step of the Lagrangian parameter in each iteration will increase the computational load of the algorithm. Therefore, it is important to develop user parameters free methods.

### 4.2. A Novel Method

If the number of source signals is *N*, there will be 2*N* local maxima of negentropy, each one of which corresponds to ±*s_i_*(*n*). The FastICA algorithm cannot theoretically obtain particular desired independent sources other than those having the maximum negentropy among the sources. Furthermore, as we know, the FastICA algorithm is a local optimization algorithm which may arbitrarily converge to different local maxima from time to time because the local convergence depends on a number of factors such as the initial weight vector and the learning rate. When one desires a specific solution, the FastICA algorithm is of little use, unless the “interesting” independent source lies in the neighborhood of the initialization. Therefore, as long as we predispose the initial **w**_0_ in the neighborhood of the SOI by utilizing the spatial constraint, the algorithm will automatically converge to it. In this case, the specific initial **w**_0_ for the SOI *s_i_*(*n*) is obtained based on the maximization of the directivity pattern corresponding to the spatial information **â***_i_* as follows:

(20) $$\begin{array}{c} \max\left|w_{0}^{H}V{\hat{a}}_{i}\right|\\ s.t.{\left\|w_{0}\right\|}^{2}-1=0 \end{array}$$

The Lagrange multiplier method is adopted to obtain the optimal solution of Equation (20). The corresponding Lagrangian function is given by:

(21) $$L({\text{w}}_{0},\mu )=\left|{\text{w}}_{0}^{H}\text{V}{\hat{a}}_{i}\right|-\mu \left({\left\|w_{0}\right\|}^{2}-1\right)$$

where *μ* is Lagrangian parameter. Let ∇**_w_**_0_ *L*(**w**_0_, *μ*) = 0, we have:

(22) $$w_{0}=V{\hat{a}}_{i}$$

Since **w**_0_ is on the unit sphere, the result is:

(23) $$w_{0}=V{\hat{a}}_{i}/\left\|V{\hat{a}}_{i}\right\|$$

The whole estimation of initial **w**_0_ for *s_i_*(*n*) via maximizing the corresponding directivity pattern does not need learning, so it is easy to obtain. In [6], Hyvarinen and Oja have shown that if the initial **w**_0_ is located in the neighborhood of **w***_i_* which is the desired projection direction to extract *s_i_*(*n*), the learning process will automatically converge to *s_i_*(*n*). Therefore, the one-unit and symmetric algorithm with purpose-designed initialization under the spatial constraint are summarized in Algorithms 3 and 4 respectively. We refer them to as Alg 3 and Alg 4 respectively in the later analysis for simplicity.


**Algorithm 3.** The one-unit version of the cICA algorithm with the purpose-designed initialization.
**Initialization**Whitened the observation data **x** to give **z** = **Vx**;**for**
*p* = 1, …, *l*Compute the specific initial weight vector 
$w_{p}^{\left(0\right)}$ corresponding the spatial constraint, that is 
${\text{w}}_{p}^{\left(0\right)}=\text{V}{\hat{a}}_{p}/\left\|V{\hat{a}}_{p}\right\|$.**Iteration**At the i^th^ iteration for obtaining **w***_p_*,
${\text{w}}_{p}^{\left(i\right)}\leftarrow \text{E}\left\{\text{z}{\left({\left(w_{p}^{\left(i-1\right)}\right)}^{H}z\right)}^{*}g\left({\left|{\left(w_{p}^{\left(i-1\right)}\right)}^{H}z\right|}^{2}\right)\right\}-\left[\text{E}\{g\left({\left|{\left(w_{p}^{\left(i-1\right)}\right)}^{H}z\right|}^{2}\right)+{\left|{\left(w_{p}^{\left(i-1\right)}\right)}^{H}z\right|}^{2}g^{\prime }\left({\left|{\left(w_{p}^{\left(i-1\right)}\right)}^{H}z\right|}^{2}\right)\right]w_{p}^{\left(i-1\right)}$
$w_{p}^{\left(i\right)}\leftarrow w_{p}^{\left(i\right)}-\sum_{j=1}^{p-1}w_{j}^{H}w_{p}^{\left(i\right)}w_{j}$
$w_{p}^{\left(i\right)}\leftarrow w_{p}^{\left(i\right)}/\left\|w_{p}^{\left(i\right)}\right\|$**Termination**The iteration is terminated when the relative change 
$\left\|w_{p}^{\left(i\right)}-w_{p}^{\left(i-1\right)}\right\|$ is less than a specified tolerance.**end for**



**Algorithm 4.** The symmetric version of the cICA algorithm with the purpose-designed initialization.
**Initialization**Whitened the observation data **x** to give **z** = **Vx**;Compute the specific initial matrix 
$W^{\left(0\right)}=\left[w_{1}^{\left(0\right)},\cdots ,w_{l}^{\left(0\right)}\right]$ corresponding the spatial constraints with 
$w_{p}^{\left(0\right)}=V{\hat{a}}_{p}/\left\|V{\hat{a}}_{p}\right\|p=1,\cdots ,l$**Iteration**At the i^th^ iteration for obtaining **W**,**for**
*p* = 1, …, *l*
${\text{w}}_{p}^{\left(i\right)}\leftarrow \text{E}\left\{\text{z}{\left({\left(w_{p}^{\left(i-1\right)}\right)}^{H}z\right)}^{*}g\left({\left|{\left(w_{p}^{\left(i-1\right)}\right)}^{H}z\right|}^{2}\right)\right\}-\left[\text{E}\{g\left({\left|{\left(w_{p}^{\left(i-1\right)}\right)}^{H}z\right|}^{2}\right)+{\left|{\left(w_{p}^{\left(i-1\right)}\right)}^{H}z\right|}^{2}g^{\prime }\left({\left|{\left(w_{p}^{\left(i-1\right)}\right)}^{H}z\right|}^{2}\right)\right]w_{p}^{\left(i-1\right)}$**end for**
$W^{\left(i\right)}\leftarrow {\left(W^{\left(i\right)}{\left(W^{\left(i\right)}\right)}^{H}\right)}^{-1/2}W^{\left(i\right)}$**Termination**The iteration is terminated when the relative change ‖**w**^(^*^i^*^)^ − **w**^(^*^i^*^−1)^‖ is less than a specified tolerance.


In summary, our new method is similar to the FastICA algorithm, including whitening, choosing an appropriate non-quadratic for contrast function, as well as the learning process for the optimization problem except for the initialization of the extracting vectors. The following theorem indicates the fact that the specific initialization obtained by Equation (23) can guarantee the learning process to the desired local maxima under certain condition.

Theorem 1: If the spatial constraint satisfies the AS4, that is to say Equation (8) comes into existence, the Alg 3 and Alg 4 must converge to the desired signal corresponding to the spatial constraint automatically.

#### Proof: See Appendix II

The theorem shows that higher accuracy of the information about the DOA is required to dispose the initial vector in the neighborhood of the solution when the non-gaussianity of the SOI is weak. In general, most communication signals are sub-Gaussian signals. Therefore, the requirement of the prior information about the DOA of the SOI is not very strict, which can be found in Section 5 by simulation.

As we know, the conventional beamforming (CBF) technique can extract the signal in the desired direction and reject all other signals in other directions, however, there are two major drawbacks: (1) the “uninteresting” source signals out of the beam can only be suppressed to some extent by the sidelobes while some “uninteresting” source signals in the beam cannot be removed, both of which are influenced by the spatial resolution; (2) The *a priori* information about the DOAs for the desired signals should be as accurate as possible. While the proposed algorithms (Alg 1–4) relax the accuracy requirement for the spatial information and may achieve better performance since they exploit the independence property of the source signals.

On the other hand, the Alg 3 and Alg 4 have obvious advantages over the conventional cICA method (*i.e.*, Alg 1 and Alg 2) in the computation load since the update of Lagrangian parameter is not involved in the learning process. Furthermore, the Alg 3 and Alg 4 are more reliable and stable due to the fact that they are user parameters free algorithms without choosing an approximate parameter measuring the corresponding constraint. We can easily see that the Alg 2 reduces to the Alg 1 and the Alg 4 reduces to the Alg 3 when there is only one SOI. The comparison results will be demonstrated in Section 5 by simulation.

## 5. Simulation Experiments

To evaluate the performance of the proposed algorithm, we adopt the average signal to interference ratio (SIR). SIR in decibels can be defined as follows:

(24) $$\text{SIR}(dB)=10\log_{10}\left(\frac{E\left\{s^{2}(n)\right\}}{E\left\{{\left(s(n)-\hat{s}(n)\right)}^{2}\right\}}\right)$$

### Experiment 1

The performance of the proposed algorithm and comparison with the FastICA algorithm and the MVDR algorithm.

In this experiment, we used four QPSK modulated signals with the carrier frequencies 12, 12.01, 12.02, 12.03 MHz, and the symbol rates 1, 2, 3, 4 Mbps, respectively. The Directions of Arrival (DOAs) of the sources were set 10^0^, 30^0^, 50^0^, and 70^0^. The signals are received by a uniform linear array with four sensors without considering the imperfect array calibration. Without loss of generality, we assume the signal with the carrier frequency 12 MHz and DOA 10^0^ to be the SOI. We chose a random initialization of **w**, *η*_1_ = 1, *η*_2_ = 0.01 and the initialization of Lagrange parameter *λ* = 10 in the Alg 1, while the threshold *γ* was set 2. In addition, the non-quadratic function $G(u)=\sqrt{0.1+u}$ was used in the adaptation of **w** in both of the Alg 1 and Alg 3. More selection for *G*(*u*) can be found in [6]. The experiment was repeated 100 times with fixed length of data samples 12,500. The following experiments will exploit the same scenario and the parameter settings without extra statements. Moreover, the FastICA algorithm identified the SOI after separating all the source signals and the extracting vector corresponding to the desired signal can be obtained by:

(25) $$\begin{array}{cc} \hat{i}=\arg\underset{k}{\max}\left|{\hat{w}}_{k}V{\hat{a}}_{i}\right| & k=1,\cdots ,N \end{array}$$

where ***ŵ****_k_* (*k* = 1, …, *N*) is the columns of the separating matrix estimated by the FastICA algorithm.

**Figure 1.**
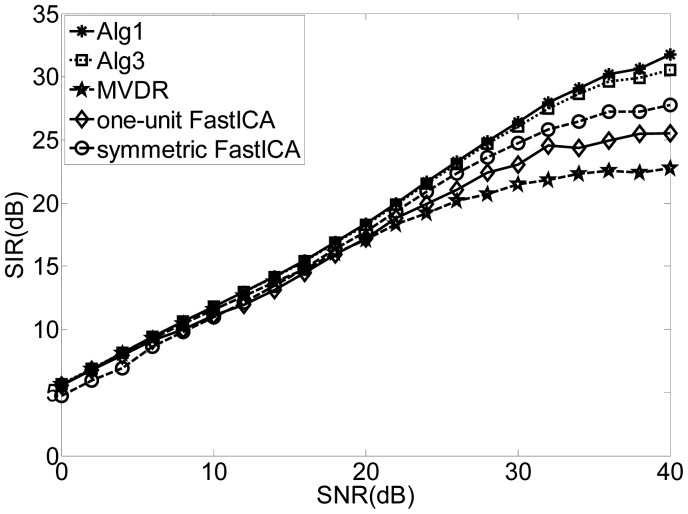
Comparison of the performance by using the algorithm of the Alg 1, Alg 3, MVDR, one-unit FastICA and symmetric FastICA.

Figure 1 shows the SIRs for different input SNRs by using Alg 1, Alg3, MVDR and the one-unit and symmetric FastICA algorithm. The SIRs of the “interesting” signals of the FastICA algorithm are worse than those of Alg 1 and Alg 3 since the FastICA algorithm attempts to separate all the other “uninteresting” components before identifying the desired signal. Again, the proposed algorithms outperform the conventional beamforming technique as our technique utilizes the independence between the source signals while the beamforming technique suffers from the leakage or cross-talk problem.

### Experiment 2

The performance comparisons among the algorithm of Alg 1, Alg 3 and MVDR with the error of the spatial constraint.

There are various factors influencing the accuracy of the spatial constraint and in this experiment we consider the following two types: (1) error of the DOA of the desired signal; (2) amplitude and phase mismatch of the array element.

**Figure 2.**
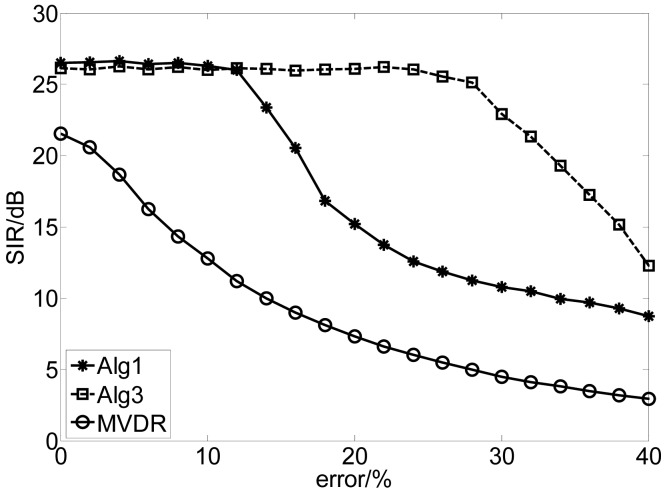
Comparison of the performance *versus* the error of DOA by using different algorithms.

The distorted DOA of the desired signal covered from 10^0^ to 12^0^, spaced with 0.1^0^ and the error of the DOA can be expressed by |*θ* − *θ_true_*| × 100/*θ_true_* (%). We ran this experiment 100 times for each value of error with fixed length of samples 12,500 when the SNR is 30 dB. The results are depicted in Figure 2. The results show that the proposed method is not sensitive to the error of DOA while the performance of the MVDR algorithm attenuates quickly as the increase of the error due to the fact that our algorithms combine the independence between the source signals and the spatial information of the SOI. Moreover, Alg 3 performs better than Alg 1 due to the different usage of the spatial information. In Alg 1, the spatial constraint has been incorporated into the learning process to guarantee the algorithm to converge to the desired solution, while in Alg 3, the spatial information is utilized to select the specific initial vector of the extracting vector. Therefore, the requirement of the accuracy of the spatial information for Alg 3 is less strict than that for Alg 1.

Let Γ be an *M* × *M* diagonal array error matrix defined as Γ = *diag*{*α*_1_*e*^−^*^jβ^*^1^, …, *α_M_e*^−^*^jβM^*} where *α_m_* and *β_m_* are the gain and the phase of the m^th^ element. We assume that *α_m_* and *β_m_* (*m* = 1, …, *M*) are independent and Gaussian random variables with *μ_α_* = 1, *μ_β_* = 0, *σ_α_* and *σ_β_*, where *μ_α_*, *μ_β_*, *σ_α_* and *σ_β_* are the mean value and the standard deviation of *α_m_* and *β_m_* (*m* = 1, …, *M*), respective. Thus, the mixing matrix defined in Equation (1) can be rewritten as:

(26) x(n)=ΓAs(n)+v(n)

**Figure 3.**
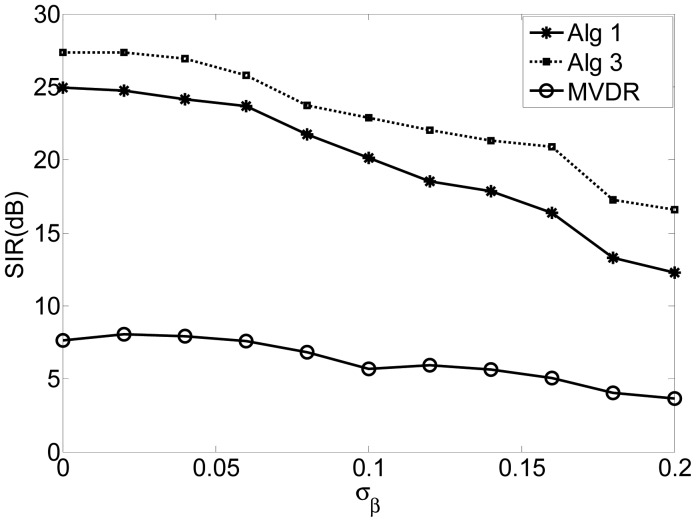
Comparison of the performance *versus* the variance of the amplitude and phase by using different algorithms.

In this experiment, *σ_α_* is assumed to be 0.01 and *σ_β_* varies from 0 to 0.2, spaced with 0.02. Figure 3 shows the SIR of the algorithms of Alg 1, Alg 3 and MVDR with respect to the standard deviation of the phase errors (*σ_β_*). The results indicate that the proposed algorithms perform more robustly than the MVDR algorithm for a wide range of phase error. Again, Alg 3 outperforms Alg 1.

The requirement of the accuracy of the spatial information for the proposed algorithm is related to various factors including the “non-Gaussianity” of the source signals and the mixing vectors corresponding to other “uninteresting” signals, as stated in Theorem 1.

### Experiment 3

The selection principle of the threshold *γ* in spatial constraint in the algorithm of Alg 1 and Alg 2.

**Figure 4.**
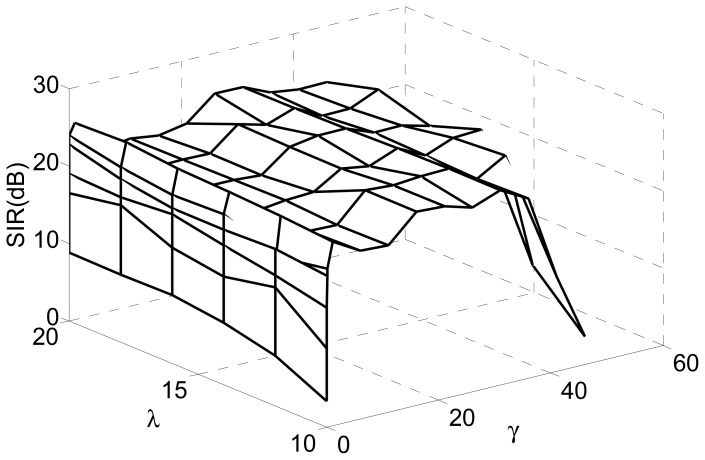
SIRs *versus* varying thresh *γ* and varying Lagrange parameter *λ*.

In this experiment, we investigated the selection principle and established the suitable range for the threshold by simulations. We changed the value of the threshold from 0.01 to 50 for fixed Lagrange parameter *λ* covering from 10 to 20. The SIRs are displayed in Figure 4, where the x-axis and y-axis represent the varying threshold and Lagrange parameter, respectively.

**Figure 5.**
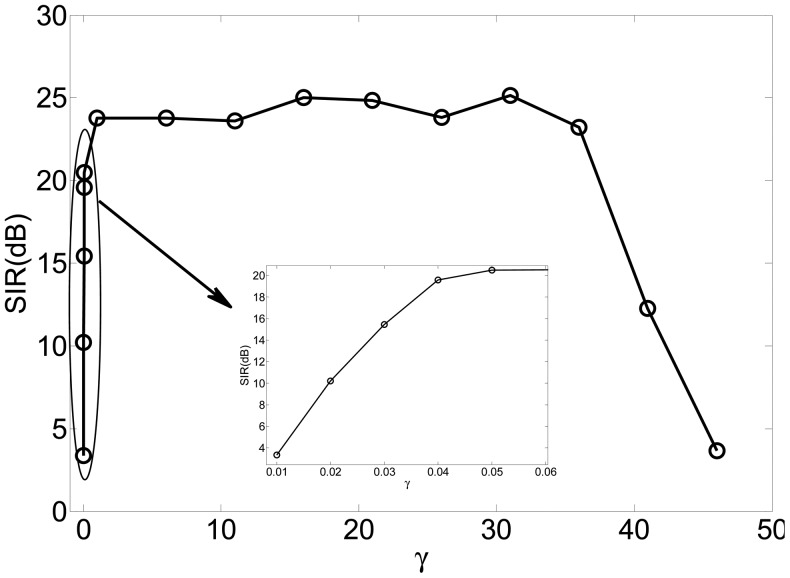
SIRs *versus* varying threshold *γ* when *λ* = 10.

**Figure 6.**
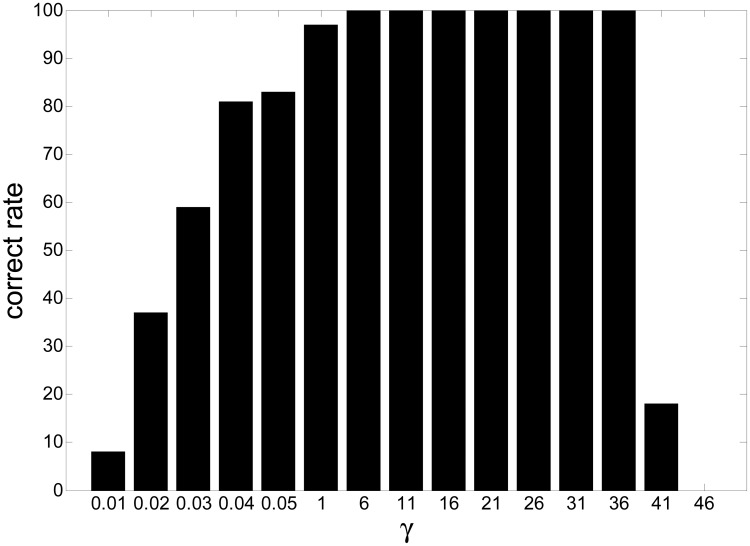
The rate of “correct” extractions *versus* varying threshold *γ*.

Figure 5 is the slice results of Figure 4 when *λ* = 10, and Figure 6 gives the rate of “correct” extraction by setting different thresholds. The results indicate that the reasonable domain for the threshold *γ* in *J*_2_(**w**) should be *γ* ∈ (1, 36;. The results demonstrate the key role of the threshold in Alg 1 and Alg 2. If it is beyond some limit, the output component may be unstable to produce any desired signal because the corresponding constraint *J*_2_(**w**) causes the learning process of that neuron to become unpredictable. If it is too small, the constraint will fail to guide the learning process to converge to the desired solution, since the spatial information of other source signals besides the SOIs may satisfy the constraint *J*_2_(**w**). Consequently, the algorithm of Alg 1 and Alg 2 cannot produce the desired solution due to the improper parameter selection, which perhaps needs a great deal of trial in different application and will not be a problem by utilizing the algorithm of Alg 3 and Alg 4.

### Experiment 4

The comparison of the consuming time between the proposed algorithm and the FastICA algorithm.

**Figure 7.**
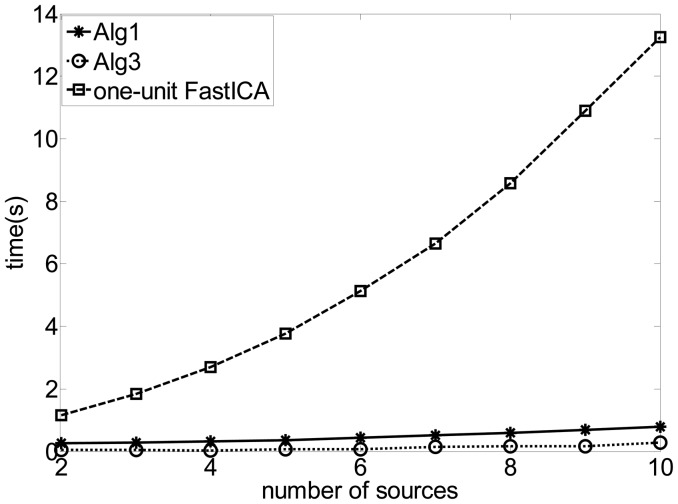
Comparison of the performance in the case of different number of the sources.

In this experiment, we added six QPSK signals with the carrier frequencies from 12.04 to 12.09 MHz spaced with 0.01 MHz. The symbol rates are all 5 Mbps and the DOAs of all the source signals were set −80^0^, −60^0^, −40^0^, −25^0^, −10^0^, 0^0^, 15^0^, 35^0^, 55^0^ and 75^0^ respectively. Figure 7 gives the comparison of the time consumed by the different algorithms with different number of source signals through the Matlab execution. The results show that the proposed algorithm runs much faster than the FastICA algorithm in extracting the SOI, because the FastICA algorithm needed redundant computation in estimating unnecessary signals. For the FastICA algorithm, the larger the number of the source signals is, the larger the computation load will be. Consequently, much more time is necessary for extracting the SOI, while the time consumed by the proposed algorithm still maintained. Furthermore, the result also indicates that Alg 1 consumes more time than Alg 3 due to the fact that the update of the Lagrangian parameter and the calculation of the inequality constraint are involved in the learning process of Alg 1, both of which makes the algorithm more complex and slows down the convergence of **w**.

### Experiment 5

Extraction of a subset of SOIs by the algorithm of Alg 1, Alg 2, Alg 3 and Alg 4.

This experiment exploited the same scenario and the same parameters as in Experiment 1 except that there were two desired signals with the DOA 10^0^ and 30^0^ to be extracted. The measurements indicating the quality of the extraction of the SOIs by using different algorithms are given in Table 1. We should note that the proposed deflationary approach, *i.e.*, Alg 1 or Alg 3, has an inherent error accumulation problem and lower convergence speed. Since we proceed from one phase to the other without complete convergence due to the finite number of iteration existence of noise and nonlinear factors, the assumption about the elimination of previous sources will fail and this will create extra noise effect for the following stage, which will accumulate throughout the multiple stages of the algorithm.

## 6. Discussion and Conclusions

In this manuscript, the authors have extended current work in the cICA framework. Spatial constraints/references are associated with sensor projections, whose location in the source mixture model is specified a priori. We incorporated the spatial constraint into the ICA model in different ways and derived two types of iterative algorithms in order to extract the “interesting” signal from the instantaneous mixture while discard other “uninteresting” sources automatically. The first class of iterative algorithms, *i.e.*, Alg 1 and Alg 2, combines the spatial constraints and the gradient descent method, and the second class of iterative algorithms, *i.e.*, Alg 3 and Alg 4, incorporates the spatial constraints into the initialization of the extracting vectors by maximizing the corresponding directivity pattern. The experimental results showed the advantages and superiority of our method compared to the previous methods. Compared with the ICA method, our algorithms perform separation and selection of the SOI simultaneously and eliminate the need for complex post-processing to detect and identify the SOI. Compared with the beamforming techniques, our methods are less sensitive to the considerable error in the DOAs of the desired signals, as well as other factors influencing the accuracy of the sensor projections, such as amplitude and phase mismatch, *etc.* Furthermore, Alg 1 and Alg 2 outperform Alg 3 and Alg 4 in both computation complexity and robustness since no extra parameters such as Lagrangian parameter and threshold are involved in the learning process, which makes the algorithm simpler and more reliable and helps to improve the speed of extraction.

Overall, the application of the cICA method has met with considerable success, especially in EM brain signal analysis, ECG/EMG signal extraction, *etc.* and it demonstrates great potential in the field of cooperative/non-cooperative communication signal processing. In fact, the cICA method can be considered as very closely related to the method called semi-blind source separation/extraction. There are numerous advantages of the cICA technique over the previous approaches: it produces only the desired independent sources and facilitates subsequent applications; the computation time and storage requirements are reduced; and the incorporation of the *a priori* information improves the quality and accuracy of the separation of the interested components or convergence speed. cICA as applied in the previous literature with temporal constraint, *i.e.*, reference signals or smoothness property, results in a useful technique for the fast and efficient extraction of the desired signals with the corresponding constraint from multichannel recordings, while the spatial constraint is exploited in our manuscript. Furthermore, when the *a priori* information mentioned in the previous literature is not available, new constraints should be explored. Moreover, different constraints can be incorporated in the cICA framework in different ways, including inequality constraints as well as specific initialization described in this manuscript. In conclusion, we need to answer the following two questions when dealing with the cICA problem.

- Which types of the *a priori* information can be exploited in the cICA problem?
- How can the *a priori* information be exploited in the cICA problem?

Additional work is being carried out to explore new features to validate the algorithm's output automatically and a new manuscript concerned about the overall description on the cICA framework answering the above two questions is being prepared by the authors.

## Figures and Tables

**Table 1.** Comparison of the performance of extracting two sources of interest by using the algorithm of Alg 1, Alg 3, Alg 3 and Alg 4.

**Algorithm**	**Output**	**SIR(dB)**
Alg 1	S1	28.9574
	S2	26.3267
Alg 2	S1	27.5611
	S2	27.8531
Alg 3	S1	27.6955
	S2	25.4508
Alg 4	S1	27.5901
	S2	27.9003

## Appendix I Derivation of Equation (19)

The first-order derivative of *J*_1_(**w**) is calculated, as follows:

(27) $$\nabla_{\text{w}}J_{1}(\text{w})=\frac{\partial J_{1}(\text{w})}{\partial {\text{w}}^{*}}=\text{E}\left\{\text{z}{\left({\text{w}}^{H}\text{z}\right)}^{*}g\left({\left|{\text{w}}^{H}\text{z}\right|}^{2}\right)\right\}$$

And the first-order derivative of *J*_2_(**w**) are calculated, as follows:

(28) $$\nabla_{\text{w}}J_{2}(\text{w})=\frac{\partial J_{2}(\text{w})}{\partial {\text{w}}^{*}}=-\text{V}{\hat{\text{a}}}_{i}{\left({\text{w}}^{H}\text{V}{\hat{\text{a}}}_{i}\right)}^{*}$$

Also, max(*J*_2_(**w**), 0) can be expressed as follows:

(29) $$\max(J_{2}(\text{w}),0)=\{\begin{array}{ll} J_{2}(\text{w}) & if\;J_{2}(\text{w})\geq 0\\ 0 & if\;J_{2}(\text{w})<0 \end{array}$$

Therefore, we can derive the following derivative

(30) $$\begin{array}{l} \nabla_{\text{w}}\max\left(J_{2}(\text{w}),0\right)=\frac{\partial }{\partial w^{*}}\max\left(J_{2}(\text{w}),0\right)\\ \;=\frac{\text{sign}\;\left(J_{2}(\text{w})\right)+1}{2}\frac{\partial J_{2}(\text{w})}{\partial {\text{w}}^{*}} \end{array}$$

Therefore, the gradient of *L*(**w**, *λ*) is calculated as follows:

(31) $$\begin{array}{l} \nabla_{\text{w}}L(\text{w},\lambda )=\nabla_{\text{w}}J_{1}(\text{w})-\lambda \nabla_{\text{w}}\max\left(J_{2}(\text{w}),0\right)\\ \;=\frac{\partial J_{1}(\text{w})}{\partial {\text{w}}^{*}}-\frac{\lambda \left(\text{sign}\left(J_{2}(\text{w})\right)+1\right)}{2}\frac{\partial J_{2}(\text{w})}{\partial {\text{w}}^{*}} \end{array}$$

Substituting Equations (27) and (28) in Equation (31), we can easily obtain

(32) $$\nabla_{\text{w}}L=E\left\{\text{z}{\left({\text{w}}^{H}\text{z}\right)}^{*}g\left({\left|{\text{w}}^{H}\text{z}\right|}^{2}\right)\right\}+\frac{\lambda }{2}\left(\text{sign}\left(J_{2}\right)+1\right)\left[\text{V}{\hat{\text{a}}}_{i}{\left({\text{w}}^{H}\text{V}{\hat{\text{a}}}_{i}\right)}^{*}\right]$$

## Appendix II Proof of Theorem 1

Without loss of generality, we assume *s_p_* is the only one SOI, **w***_p_* is the solution of extracting vector, and the initial weight of **w***_p_* is ${\text{w}}_{p}^{\left(0\right)}=\text{V}{\hat{\text{a}}}_{p}/\left\|\text{V}{\hat{\text{a}}}_{p}\right\|$. So we have

(33) $$s_{p}={\text{w}}_{p}^{H}\text{z}={\text{w}}_{p}^{H}\text{AsV}={\text{e}}_{p}^{H}\text{s}$$

(34) $${\hat{s}}_{p}={\left({\text{w}}_{p}^{\left(0\right)}\right)}^{H}\text{z}={\left({\text{w}}_{p}^{\left(0\right)}\right)}^{H}\text{VAs}={\text{q}}_{p}^{H}\left(0\right)\text{s}$$

where **e***_p_* = [0, …, *c*, …, 0]*^H^* with |*c*| = 1 and **q***_p_*(0) = (**VA**)*^H^*(**VA**)**A**^−1^***â****_p_* = **A**^−1^***â****_p_* = [*q_p_*_,1_, …, *q_p,N_*]*^H^* with the unit-norm constraint ‖**q***_p_*(0)‖ = 1. Now, we just need to proof **q***_p_* must converge to **e***_p_*. Here, we use kurtosis as the measurement of non-Gaussianity instead of negentropy and gradient learning algorithm as the optimization method for simplicity, since Newton-like method has the same extreme value which has been shown in [6].

According to 0, kurtosis has linearity property that

(35) $$\text{kurt}\left({\text{q}}_{p}^{H}\text{s}\right)=\sum_{i=1}^{N}{\left|q_{p,i}\right|}^{4}\text{kurt}\left(s_{i}\right)$$

And the gradient can be calculated as

(36) $$\frac{\partial \text{kurt}\left({\text{q}}_{p}^{H}\text{s}\right)}{\partial {\text{q}}_{p}^{*}}={\left[2q_{p,1}{\left|q_{p,1}\right|}^{2}\text{kurt}\left(s_{1}\right),2q_{p,2}{\left|q_{p,2}\right|}^{2}\text{kurt}\left(s_{2}\right),\cdots ,2q_{p,N}{\left|q_{1,N}\right|}^{2}\text{kurt}\left(s_{N}\right)\right]}^{T}$$

Equating the direction of gradient with new **q***_p_*, we have

(37) $$\begin{array}{ccc} {\text{q}}_{p}\propto \frac{\partial \text{kurt}\left({\text{q}}_{p}^{H}\text{s}\right)}{\partial {\text{q}}_{p}^{*}} & \;\text{and}\; & {\text{q}}_{p}={\text{q}}_{p}/\left\|{\text{q}}_{p}\right\| \end{array}$$

Therefore, the learning process of the elements of **q***_p_* can be written as

(38) $$\begin{array}{cc} q_{p,i}(k+1)=c(k)\text{kurt}(s_{p})q_{p,i}(k){\left|q_{p,i}(k)\right|}^{2} & i=1,\cdots ,N \end{array}$$

where *c*(*k*) is a constant which makes the norm of **q***_p_*(*k* + 1) equal unity. Then we have

(39) $$\left|\frac{q_{p,p}(k+1)}{q_{p,i}(k+1)}\right|=\left|\frac{c(k)c^{3}(k-1)\cdots c^{3^{k}}(0)\text{kur}t^{\frac{3^{k}-1}{2}}(s_{p})q_{p,p}^{3^{k}}(0)}{c(k)c^{3}(k-1)\cdots c^{3^{k}}(0)\text{kur}t^{\frac{3^{k}-1}{2}}(s_{i})q_{p,i}^{3^{k}}(0)}\right|={\left|\frac{\text{kurt}(s_{p})}{\text{kurt}(s_{i})}\right|}^{\frac{3^{k}-1}{2}}\left|\frac{{\tilde{\text{a}}}_{p}^{H}{\hat{\text{a}}}_{p}}{{\tilde{\text{a}}}_{i}^{H}{\hat{\text{a}}}_{p}}\right|p\neq i$$

Case 1: |*kurt*(*s_p_*)|≥|*kurt*(*s_i_*)| and $\left|\frac{{\tilde{\text{a}}}_{p}^{H}{\hat{\text{a}}}_{p}}{{\tilde{\text{a}}}_{i}^{H}{\hat{\text{a}}}_{p}}\right|>1$
  (40) $$\left|\frac{q_{p,p}(k+1)}{q_{p,i}(k+1)}\right|>{\left|\frac{\text{kurt}(s_{p})}{\text{kurt}(s_{i})}\right|}^{\frac{3^{k}-1}{2}}>1$$
Case 2: |*kurt*(*s_p_*)|<|*kurt*(*s_i_*)|
  (41) $$\begin{array}{l} \left|\frac{q_{p,p}(k+1)}{q_{p,i}(k+1)}\right|={\left|\frac{\text{kurt}(s_{p})}{\text{kurt}(s_{i})}\right|}^{\frac{3^{k}}{2}}{\left|\frac{\text{kurt}(s_{i})}{\text{kurt}(s_{p})}\right|}^{\frac{1}{2}}\left|\frac{{\tilde{\text{a}}}_{p}^{H}{\hat{\text{a}}}_{p}}{{\tilde{\text{a}}}_{i}^{H}{\hat{\text{a}}}_{p}}\right|\\ \;>{\left|\frac{\text{kurt}(s_{p})}{\text{kurt}(s_{i})}\right|}^{\frac{3^{k}}{2}}{\left|\frac{\text{kurt}(s_{i})}{\text{kurt}(s_{p})}\right|}^{\frac{3^{k}}{2}}{\left|\frac{\text{kurt}(s_{i})}{\text{kurt}(s_{p})}\right|}^{\frac{1}{2}}\\ \;={\left|\frac{\text{kurt}(s_{i})}{\text{kurt}(s_{p})}\right|}^{\frac{1}{2}}>1 \end{array}$$

Therefore, for any *k* in the learning process, the following inequality always holds

(42) $$\begin{array}{cc} \left|\frac{q_{p,p}(k+1)}{q_{p,i}(k+1)}\right|>1 & p\neq i \end{array}$$

Since [6,27] has proved that the FastICA algorithm will converge and once the algorithm converges, only one element of **q***_p_* equals *c* with |*c*| = 1, and according to Equation (42) **q***_p_* must converge to **e***_p_*. This completes the proof.

## References

[1] Smith D., Lukasiak J., Burnett I. (2006). An analysis of the limitations of blind signal separation application with speech. Signal Proc..

[2] Jiménez-Hernández H. (2010). Background subtraction approach based on independent component analysis. Sensors.

[3] Lee J., Park K.L., Lee K.J. (2005). Temporally constrained ICA-based fetal ECG separation. Electron. Lett..

[4] Yang X.N., Yao J.L., Jiang D., Zhao L. Performance analysis of the FastICA algorithm in ICA-Based Co-Channel Communication System.

[5] Johnson D.H., Dudgeon D.E. (1992). Array Signal Processing: Concepts and Techniques.

[6] Hyvarinen A., Karhunen J., Oja E. (2001). Independent Component Analysis.

[7] Saruwatari H., Takeda K., Itakura F., Nishikawa T., Shikano K. (2003). Blind source separation combining independent component analysis and beamforming. EURASIP J. Appl. Signal Proc..

[8] Saruwatari H., Takeda K., Nishikawa T., Itakura F., Shikano K. (2006). Blind source separation based on a fast-convergence algorithm combining ica and beamforming. IEEE Trans. Audio Speech Lang. Proc..

[9] Parra L.C., Alvino C.V. (2002). Geometric source separation: Merging convolutive source separation with geometric beamforming. IEEE Trans. Speech Audio Proc..

[10] Lu W., Rajapakse J.C. (2005). Approach and applications of constrained ICA. IEEE Trans. Neural Networks.

[11] Lu W., Rajapakse J.C. (2006). ICA with reference. Neurocomputing.

[12] Lu W., Rajapakse J.C. (2003). Eliminating indeterminacy in ICA. Neurocomputing.

[13] James C.J., Gibson O.J. (2003). Temporally constrained ICA: An application to artifact rejection in electromagnetic brain signal analysis. IEEE Trans. Biomed. Eng..

[14] Mitianoudis N., Stathaki T., Constantinides A.G. (2007). Smooth signal extraction from instantaneous mixtures. IEEE Signal Proc. Lett..

[15] Hesse C.W., James C.J. (2005). The FastICA algorithm with spatial constraints. IEEE Signal Proc. Lett..

[16] Bell A.J., Sejnowski T.J. (1995). An information-maximization approach to blind separation and blind deconvolution. Neural Comput..

[17] Cardoso J.F., Souloumiac A. (1993). Blind beamforming for non-Gaussian signals. IEE Proc..

[18] Hyvarinen A. (1999). Fast and robust fixed-point algorithms for independent component analysis. IEEE Trans. Neural Networks.

[19] Bingham E., Hyvarinen A. (2000). A fast fixed-point algorithm for independent component analysis of complex valued signals. Int. J. Neural Syst..

[20] Douglas S.C. (2007). Fixed-Point algorithms for the blind separation of arbitrary complex-valued non-gaussian signal mixtures. EURASIP J. Adv. Signal Proc..

[21] Li X.L., Adali T.L. (2010). Independent component analysis by entropy bound minimization. IEEE Trans. Signal Proc..

[22] Javidi S., Took C.C., Mandic D.P. (2011). Fast independent component analysis algorithm for quaternion valued signals. IEEE Trans. Neural Networks.

[23] Mi J.X., Gui J. (2010). A method for ICA with reference signals. Adv. Intell. Comput. Theor. Appl. Asp. Artif. Intell..

[24] Nocedal J., Wright S.J. (1999). Numerical Optimization.

[25] Zarzoso V., Comon P. (2007). Comparative speed analysis of fastica. Lect. Notes Comput. Sci..

[26] Zarzoso V., Comon P. (2010). Robust independent component analysis by iterative maximization of the kurtosis contrast with algebraic optimal step size. IEEE Trans. Neural Networks.

[27] Shen H., Kleinsteuber M., Hüper K. (2008). Local convergence analysis of fastica and related algorithms. IEEE Trans. Neural Networks.

